# Nutrient availability limits biological production in Arctic sea ice melt ponds

**DOI:** 10.1007/s00300-017-2082-7

**Published:** 2017-03-01

**Authors:** Heidi Louise Sørensen, Bo Thamdrup, Erik Jeppesen, Søren Rysgaard, Ronnie Nøhr Glud

**Affiliations:** 1 Department of Biology, Nordic Centre for Earth Evolution (NordCEE), University of Southern Denmark (SDU), Campusvej 55, 5230 Odense, Denmark; 2 Greenland Climate Research Centre (GCRC), Greenland Institute of Natural Resources, Kivioq 2, 3900 Nuuk, Greenland; 3 Department of Bioscience, Aarhus University, Vejlsøvej, 25, 8600 Silkeborg, Denmark; 4 Sino-Danish Centre for Education and Research (SDC), The University of the Chinese Academy of Sciences (UCAS), Beijing, 100190 China; 5 Department of Environment and Geography, Centre for Earth Observation Science, University of Manitoba, Winnipeg, MB R3T 2N2 Canada; 6 Department of Geological Sciences, University of Manitoba, Winnipeg, MB R3T 2N2 Canada; 7 Arctic Research Centre, Aarhus University, 8000 Aarhus, Denmark; 8 Scottish Association for Marine Science, Scottish Marine Institute, Oban, PA37 1QA UK

**Keywords:** Arctic, Sea ice melt ponds, Nutrients, Primary production, Bacterial production, Grazers and higher trophic levels

## Abstract

Every spring and summer melt ponds form at the surface of polar sea ice and become habitats where biological production may take place. Previous studies report a large variability in the productivity, but the causes are unknown. We investigated if nutrients limit the productivity in these first-year ice melt ponds by adding nutrients to three enclosures ([1] PO_4_^3−^, [2] NO_3_^−^, and [3] PO_4_^3−^ and NO_3_^−^) and one natural melt pond (PO_4_^3−^ and NO_3_^−^), while one enclosure and one natural melt pond acted as controls. After 7–13 days, Chl *a* concentrations and cumulative primary production were between two- and tenfold higher in the enclosures and natural melt ponds with nutrient addition compared with their respective controls, with the largest increase occurring in the enclosures. Separate additions of PO_4_^3−^ and NO_3_^−^ in the enclosures led to intermediate increases in productivity, suggesting co-limitation of nutrients. Bacterial production and the biovolume of ciliates, which were the dominant grazers, were positively correlated with primary production, showing a tight coupling between primary production and both microbial activity and ciliate grazing. To our knowledge, this study is the first to ascertain nutrient limitation in melt ponds. We also document that the addition of nutrients, although at relative high concentrations, can stimulate biological productivity at several trophic levels. Given the projected increase in first-year ice, increased melt pond coverage during the Arctic spring and potential additional nutrient supply from, e.g. terrestrial sources imply that biological activity of melt ponds may become increasingly important for the sympagic carbon cycling in the future Arctic.

## Introduction

Rising temperatures have reduced the extent and thickness of sea ice in the Arctic region (e.g. Serreze et al. 2000; Haas et al. 2008), leading to the replacement of most of the multiyear ice with first-year ice (Maslanik et al. 2007; Comiso 2012). These changes have increased the total areal coverage of melt ponds during the Arctic summer (Nicolaus et al. 2012; Rösel and Kaleschke 2013). The temporal evolution in melt pond coverage is mainly determined by the increasing atmospheric temperature during spring and summer, facilitating snow melting (Bursa 1963). Typically, the relative areal melt pond coverage increases at an exponential rate, peaking at 20–50% (Eicken et al. 2002), thereby strongly reducing the overall sea ice albedo (Polashenski et al. 2012; Perovich and Polashenski 2012). Hence, melt ponds enhance light availability within and below the sea ice (Nicolaus et al. 2012), which stimulates the light-limited biological productivity and can lead to early nutrient depletion in surface waters before ice brake-up (Arrigo et al. 2014). The ponds in themselves also represent a microbial habitat (Bursa 1963), but the results of the so far few available studies reflect a wide range of productivity in these melt ponds, from almost insignificant production (Mundy et al. 2011; Fernández-Méndez et al. 2015) to highly productive ponds covered by microbial mats and aggregates (Lee et al. 2011; Fernández-Méndez et al. 2014). It has been speculated that this large range in production may be associated with differences in nutrient availability (Mundy et al. 2011; Fernández-Méndez et al. 2015) and with incident UV-radiation as an additional moderator of the biological productivity (Marcoval et al. 2007; Wängberg et al. 2008). Nutrient availability is typically correlated with salinity (e.g. Mundy et al. 2011), suggesting that a substantial snow cover would reduce nutrient availability in the melt ponds as the melting of the snow progresses. In contrast, inflow, spraying, and flooding from surface sea water could likely increase nutrient availability and thus stimulate productivity. The algal community in the melt ponds can consist of fresh water species, such as *Chlamydomonas nivalis* and *Meringosphaera mediterranea* (Melnikov et al. 2002), as well as marine species, which are mainly dominated by diatom genera such as *Navicula* sp., *Nitzchia* sp., *Thalassiosira* sp., *Chaetoceros* sp. and *Melosira arctica* (Bursa 1963; Melnikov et al. 2002; Fernández-Méndez et al. 2014). The presence of diatom species is consistent with the algal community found within the interior of sea ice (e.g. Mundy et al. 2011) or right below the ice, suggesting that the melt pond community structure is mainly determined by the community occurring within the sea ice (e.g. Bursa 1963). Along with the bacterial community, these primary producers represent the foundation of ice-associated food webs, and therefore, regulate potential colonization by higher trophic levels (Bursa 1963; Lee et al. 2011; Fernández-Méndez et al. 2014). Hence, to understand the present-day Arctic sympagic carbon cycling and how the predicted increase in melt pond coverage might alter this, it is important to determine the limiting factor for primary production in melt ponds. The aim of the present study was, therefore, to experimentally determine if nutrients limit total biological production in ice melt ponds of Arctic sea ice.

## Materials and methods

### Study site and melt pond setup

**Fig. 1:**
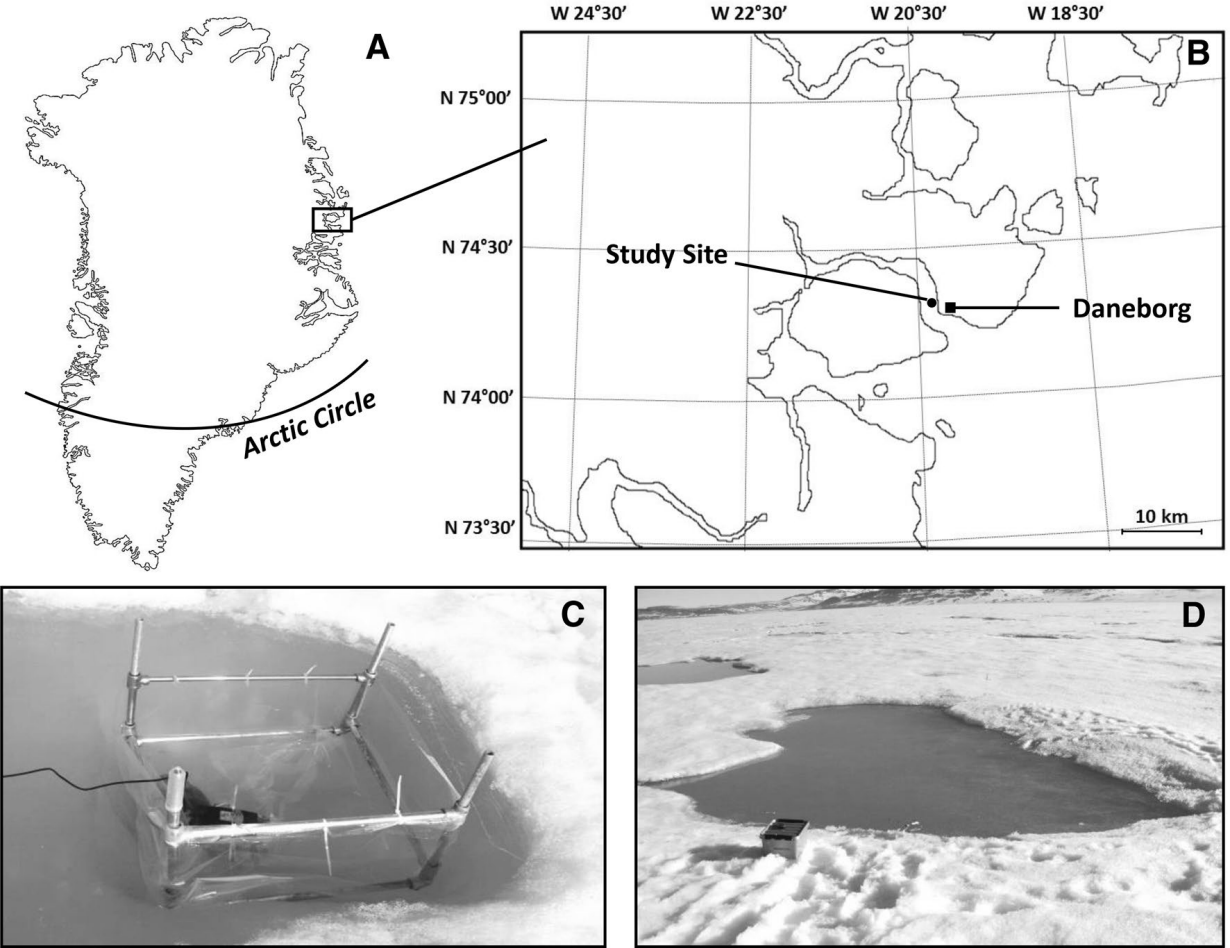
**a** Location of Young Sound on the north-east coast of Greenland and **b** the location of the study site (*filled circle*) situated approximately 2 km (74°19′39 N, 20°14′37 W) from the research station Daneborg. The figure is modified from Glud et al. (2000). **c** One of the enclosures (50 × 50 × 50 cm) submerged in a fitted hole in the sea ice and **d** one of the natural melt ponds monitored during the study (area ~ 25 m^2^)

The study was carried out during June 2014 in Young Sound (NE Greenland) (Fig. 1a), ~2 km from the coastline just outside Daneborg (Fig. 1b). At the study site, sea ice normally forms in October and gradually grows to a maximum thickness of ~150 cm in May. Hereafter the sea ice thickness declines rapidly, transitioning into a free-floating ice cover that is exported from the fjord in early July (Rysgaard et al. 1999). Melt pond formation typically starts towards the end of May, with the melt pond coverage increasing at an exponential rate towards the sea ice break-up (e.g. Rysgaard et al. 1999; Rysgaard and Glud 2007). During the 2014 sea ice melting season, the increasing melt pond coverage was estimated from daily photos of the sea ice taken from the shore, adjusting the perspective in each photo and estimating the fraction of the melt ponds using the software program ImageJ.

To maintain controlled conditions, a series of enclosures, each containing 40–55 L melt pond water, was established (Fig. 1c). The enclosures were constructed as square basins (sides 50 cm, depth 50 cm) of laminated transparent and gastight NEN/PE plastic foil (e.g. Hansen et al. 2000) supported in the corners by hollow aluminum pipes (1.2 cm i.d.), keeping the water surface exposed to the atmosphere. All enclosures were submerged in holes that were cut into the sea ice and filled to the extent where the water surface matched the sea ice surface using water sampled from one natural melt pond (the volume used comprised less than 1% of the original melt pond water volume). Submersible pumps were placed in each enclosure to avoid stratification and to partially mimic the natural wind-driven mixing in the natural melt ponds. In parallel to the enclosures, two natural melt ponds (Fig. 1d) were monitored during the study period. These initially covered an area of 25 and 130 m^2^, but the areal coverage gradually increased to twice the size at the end of the study. Incident radiation and temperature in both the atmosphere (0.5 m above the sea ice surface) and melt ponds were continuously recorded by small loggers (Hobo pendant, Onset). The loggers were cross-calibrated to a handheld light meter (WALZ ULM-500 Light Meter, WALZ spherical Micro Quantum Sensor US-SQS/L). Nutrients were added to three of the enclosures on the same day, and one was maintained as control. The following nutrient treatments (either as single or dual nutrient addition) were applied: (1) PO_4_^3−^, (2) NO_3_^−^ and (3) PO_4_^3−^ and NO_3_^−^. To avoid nutrient depletion especially in case of unintentional leakage, we decided to apply relative high nutrient concentrations. The initial concentrations of PO_4_^3−^ and NO_3_^−^ in the enclosures were therefore adjusted to ~4 and 25 µmol L^−1^, respectively, which was ~100 times higher than the in situ values. The nutrient concentrations were measured the following day, and additional nutrients were added to the enclosure with the dual nutrient addition, as the concentrations in this enclosure were below the intended concentration increase. Additionally, bromide (Br^−^) was added to a concentration of 1 mM and was used as an inert tracer to test for potential leakage and dilution of the enclosed water. In addition to the enclosures, one of the natural ponds (Fig. 1d) was treated with dual addition of PO_4_^3−^ and NO_3_^−^ to final concentrations of 7 and 40 µmol L^−1^, respectively, these being slightly higher than those employed in the enclosures due to overestimation of the melt pond volume. Given the very strong enrichment relative to in situ values, the differences in concentrations between enclosures and pond were not expected to affect the results substantially. The conditions in two of the enclosures (control and dual nutrient addition) and the two natural melt ponds were monitored over 13 days, while the remaining two enclosures (single nutrient additions) were monitored for only 7 days as these were unintentionally flooded on day 7. Due to time limitation, only 2–3 enclosures or natural melt ponds were measured per day, resulting in a time shift between the sampling of the respective enclosures and ponds. The measured variables included salinity and the concentrations of chlorophyll *a* (Chl *a*), particulate organic carbon (POC), PO_4_^3−^, NO_3_^−^ and Br^−^. In addition, rates of primary production and bacterial production were measured in subsamples during short-term incubation (see below). Furthermore, the biovolume of potential microbial grazers was determined in two of the enclosures and the two natural melt ponds at the beginning and at the end of the study. To assess statistical significance for the changes in the measured concentrations and rates, *T-tests* with a significance level of 5% were used to assess if the slopes were significantly different from 0 and if the slopes were significantly different from each other.

After the field campaign, we became aware that there was a potential for sorption of PO_4_^3−^ onto the aluminum pipes supporting the enclosures. We therefore, tested this in the laboratory by placing sections of the pipes in two beakers containing GF/C (Whatman) filtered sea water diluted to salinity 3 psu with milli-Q water, while additionally two beakers with the same water and no aluminium pipe sections acted as controls. PO_4_^3−^ and NO_3_^−^ were added to all beakers in a final concentration of 4 and 25 µmol L^−1^, respectively. The beakers were incubated on a shaking table placed in a cooling room at 5 °C and the nutrient concentrations were monitored over 3 days. Subsamples for nutrient determination were stored and analyzed as described below. Potential toxic effects of the aluminum pipes on the microbial performance in the enclosures was not investigated.

### Measurements of Chl *a*, POC, PO_4_^3−^, NO_3_^−^ and Br^−^ in enclosures and melt ponds

To extract Chl *a*, 500 mL of melt pond water was filtered onto GF/F filters (Whatman) and the filters were placed in test tubes containing 10 mL ethanol (96%). The samples were stored in darkness at −18 °C until analysis using a fluorometer (Turner TD-700 fluorometer, Turner Designs, California, USA). For POC determination, an additional 500 ml was filtered through separate pre-combusted GF/F filters (Whatman). These filters were stored frozen (−18 °C) in pre-combusted aluminum foil packages. For analysis, the filters were acidified to remove inorganic carbon and thereafter packed into tin capsules for determination of the organic carbon content using a solid phase elemental analyzer (CHN EA1108-Elemental analyzer, CARLO ERBA). For determination of PO_4_^3−^, NO_3_^−^ and Br^−^ concentrations, 50 mL of the filtrate was stored frozen (−18 °C). PO_4_^3−^ and NO_3_^−^ concentrations were measured by standard procedures as described in Grasshoff et al. (1983) and García-Robledo et al. (2014), respectively, while Br^−^ concentrations were measured using ion chromatography (IC, Dionex IC S-1500; Forster et al. 1999).

### Primary production in the enclosures and melt ponds

Primary production was measured using a modified version of the original ^14^C-incubation method described in Steeman-Nielsen (1952). The water sample was added to three transparent and one darkened 120 mL glass bottle (Hirshmann) and spiked with 200 µL NaH^14^CO_3_ (20 µCi mL^−1^). To ensure that temperature in the darkened bottles remained the same as in the transparent bottles, the darkened bottles were covered by white tape. The bottles were sealed with glass stoppers and incubated for 3–6 h. All short-term incubations were performed with the incubation bottles attached to the metal frame in the respective enclosures or placed on the bottom of the natural melt ponds (~15 cm below the ambient sea ice surface). This ensured that all short-term incubations were exposed to similar light conditions and in situ temperatures. At the end of the short-term incubations, the samples were transported in a darkened and thermo-insulated box to the laboratory within 1 h. Upon the return, the samples were filtered onto GF/F filters (Whatman), which were transferred to scintillation vials (20 mL) and stored frozen (−18 °C) until further processing at the Greenland Institute of Natural Resources, Nuuk. Here, the samples were acidified and fumed for 24 h to remove non-bound excess ^14^CO_3_ and ^14^CO_2_ prior to adding 10 mL scintillation cocktail (Ultima Gold, Perkin Elmer) to each sample (e.g. Søgaard et al. 2010). The fixed amount of ^14^C carbon of the respective samples was subsequently quantified with a scintillation counter (Liquid Scintillation Analyzer, Tri-Carb 2800TR, PerkinElmer). Primary production rates (mmol C m^−3^ day^−1^) were calculated accounting for the DIC concentration in the respective enclosure or natural melt pond at the sampling time, the discrimination factor ranging between ^12^CO_2_ and ^14^CO_2_ (1.05) of algae assimilation, a correction factor for the respiration of organic matter during the experiment (1.06; Becacos-Kontos 1965), the specific activity of the added ^14^ CO_3_ and the incubation time. The resulting rates were extrapolated to 24 h using the ratio between integrated irradiance during the incubation period versus the irradiance during 24 h of midnight sun day (e.g. Juul-Pedersen et al. 2015).

### Bacterial production measurements

Bacterial production was measured using two independent methods: ^3^H-thymidine incorporation in bacterial DNA and ^3^H-Leucine fixation during protein synthesis (Chin-Leo and Kirchman 1988). In both types of incubation, water was initially filtered through GF/C filters (Whatman) and 10 mL was transferred to each of six darkened 15 mL falcon tubes to which either ^3^H-thymidine (final concentration of 10 nM) or ^3^H-Leucine (final concentration of 20 nM) was added. For each tracer, one sample was terminated at the beginning of the short-term incubation by the addition of 1 mL of cold trichloroacetic acid (TCA, 50%) to stop biological activity. Duplicate incubations for each tracer were conducted in the enclosures and the incubations were terminated after 4–6 h. Samples were stored at 3 ± 1 °C until further processing at University of Southern Denmark. Here, the samples were filtered onto cellulose ester filters (ADVANTEC A020A047A, 0.2 µm, 47 mm), rinsing the falcon tubes with 5 mL cold TCA (5%). Following this, the filters were rinsed with 1 mL TCA (5%) seven times. The filters were transferred to scintillation vials and 10 mL of scintillation cocktail was added to each vial before the amount of fixed ^3^H-labeled substrates in each sample was quantified with the scintillation counter (Liquid Scintillation Analyzer, Tri-Carb 2910TR, PerkinElmer). Bacterial production (mmol C m^−3^ day^−1^) was calculated by converting the estimated moles of thymidine and leucine incorporated into cell biomass using the coefficients 2.09 × 10^18^ and 6.40 × 10^16^ cells mol^−1^, respectively (e.g. Kirchman and Hoch 1988) and assuming a cell-specific carbon content of 5.7 × 10^−8^ µg C cell^−1^ (Søgaard et al. 2010). Values were corrected for the specific activity of the added tracers, the incubation time and the sample volume.

### Identification and biovolume of algal species, grazers and higher trophic levels

At the beginning and end of the study, 5–10 L of sampled melt pond water was used for the identification of potential grazers and their relative abundance and biovolume. Unfortunately, two of the enclosures became submerged during the study, preventing retrieval of end samples, so only a total of five samples were investigated by this approach. Upon return to the laboratory, the water samples were fixed with Lugol (2%) and left in darkness for 24 h. During this period, the fixed organisms settled and the overlying water was subsequently removed, thereby concentrating the fixed organisms at the bottom. The concentrated samples were transferred to dark bottles and the Lugol concentration was increased to 4%. Species were subsequently taxonomically identified and biovolume (mm^3^ L^−1^) was determined using an inverted microscope (Jeppesen et al. 2002).

### Primary and bacterial production in sea ice

On two occasions (19 and 23 June 2014), sea ice cores were collected ~4 km from the main sampling site using a 9 cm in diameter ice corer (MARK II Coring system, Kovacs enterprises). Each ice core was sectioned into five slices: one surface (0–10 cm), two intermediate (40–50; 80–90 cm) and two bottom (115–125; 125–135 or 109–119; 119–129 cm) slices for determination of primary and bacterial productions. The ice was melted within 24 h in closed bottles at 4 ± 1 °C in the dark. Primary production was assessed by placing 12 × 75 mL bottles in an incubator exposed to a light gradient (light interval in PAR: 3, 22, 26, 35, 51, 63, 90, 120, 125, 191, 220 µmol photons m^−2^ s^−1^). Cooled water was continuously pumped into the incubator and maintained at a constant temperature of 4 ± 1 °C. Each incubation bottle was filled with the melted sea ice sample and spiked with 100 µL NaH^14^CO_3_ (20 µCi mL^−1^). The samples were incubated for 3–6 h and incubation was terminated by placing the bottles in a dark box prior to filtration onto GF/F filters (Whatman) as described above. Filtration was performed within 1 h after termination of the incubation. PE relations were determined for each sea ice section using curve fitting with the following function: $\text{PP}~=~{{P}_{\text{m}}}\left( 1-{{e}^{\frac{-\alpha ~{{E}_{\text{PAR}}}}{{{P}_{\text{m}}}}}} \right)$ (Platt et al. 1980), where *P*_m_ is the maximum primary production rate, α is the initial slope of the PE curve and *E*_PAR_ is the irradiance in PAR (µmol Photons m^−2^ s^−1^). Primary production rate profiles in the sea ice were estimated using the PE relations and the daily rates of incident solar radiation, applying estimated light attenuation constants for snow (9.5 m^−1^) and sea ice (3.2 m^−1^) previously measured in the area (Glud et al. 2007). The bacterial production rate of the melted sea was determined in parallel at 4 ± 1 °C using only ^3^H-thymidine incorporation as described above.

## Results

**Fig. 2:**
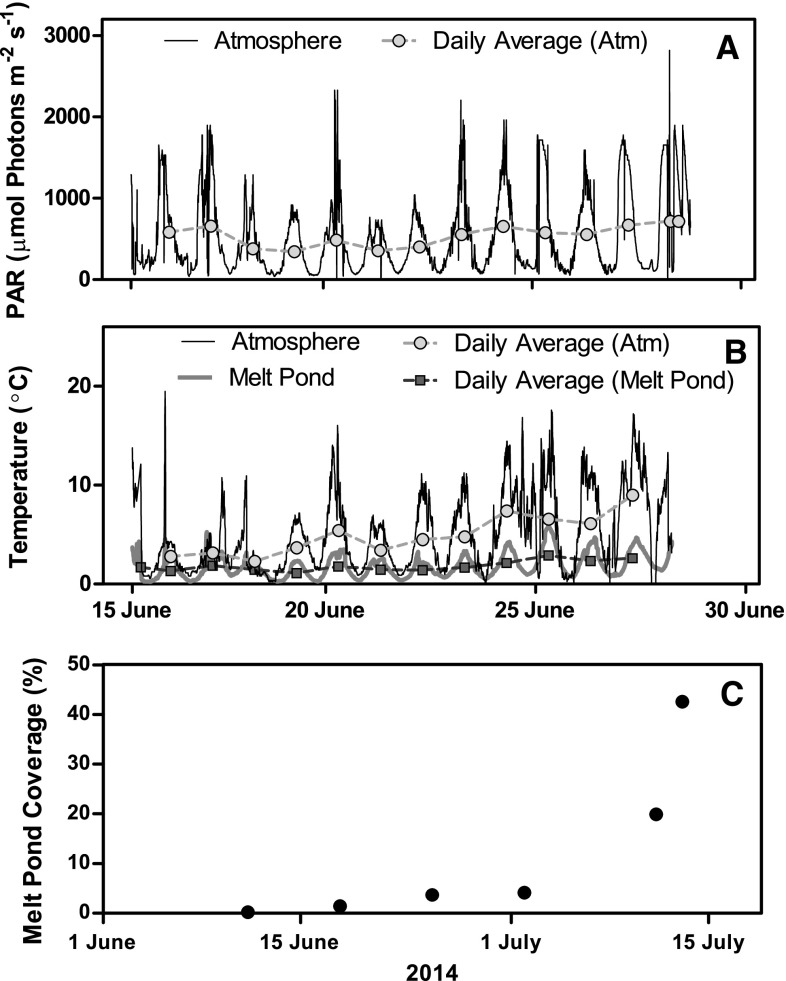
Continuous measurements of light (PAR, µmol Photons m^−2^ s^−1^) from the 15th to the 29th of June 2014 in **a** the atmosphere and **b** the corresponding temperatures (°C) measured in both the atmosphere and a melt pond. The daily averages are indicated with *filled circles*. **c** Changes in melt pond coverage from the 13th of June to the 14th of July 2014 was estimated from daily ground pictures of sea ice conditions taken from the shore at Young Sound. Photoshop was used to change the perspective of the pictures and ImageJ was used to quantify the fraction of melt ponds relative to the snow-covered ice

Incident PAR at Young Sound varied between 48 and 2818 µmol photons m^−2^ s^−1^ during the study period (15–30 June), with the daily average varying from 311 to 716 µmol photons m^−2^ s^−1^ (Fig. 2a). During the same period, the atmospheric temperature varied from −2 and occasionally reached almost 20 °C, while daily average temperatures ranged from 2 to 7 °C (Fig. 2b). The diel variation in atmospheric temperature was reflected, but dampened, in the water of the melt ponds, and the daily averages ranged between 1 and 3 °C (Fig. 2b). At the beginning of the study, the snow cover was ~20 cm, but increasing temperatures led to its gradual reduction and to melt pond formation in the beginning of June. Melt pond coverage increased from ~1% on 11 June to ~40% by 14 July (Fig. 2c). By 15 July, the remaining sea ice was exported from Young Sound by the tide and wind.

### Melt pond nutrient additions

**Fig. 3:**
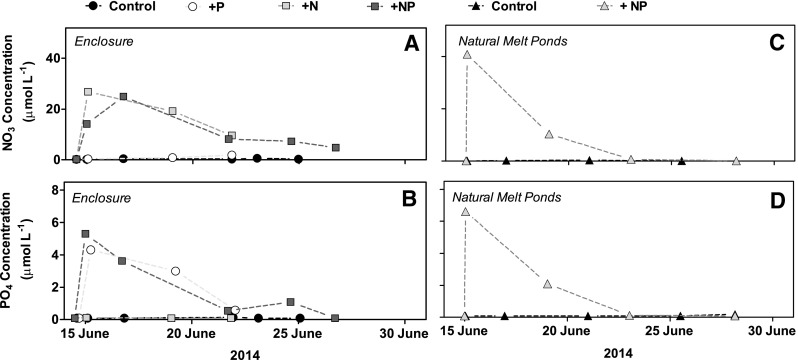
Concentrations of **a** NO_3_^−^ and **b** PO_4_^3−^ in the enclosures measured at each time point during the study. Additional NO_3_ was added to the enclosures with dual nutrient addition to adjust the concentration. Concentrations of **c** NO_3_^−^ and **d** PO_4_^3−^ in the natural melt ponds

**Fig. 4:**
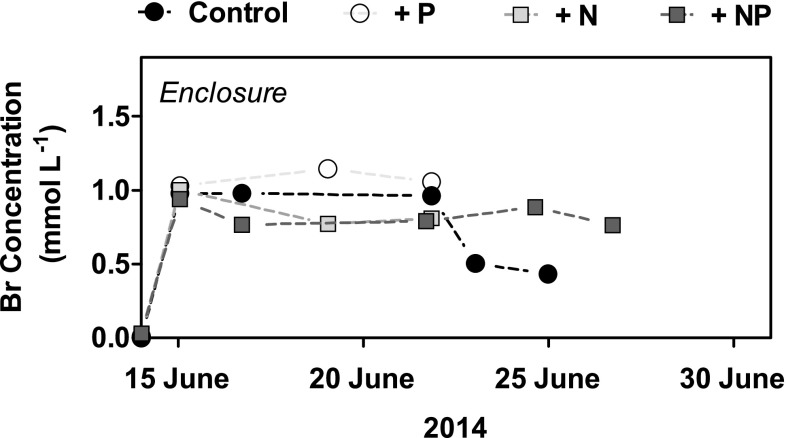
**a** Br^−^ concentration in enclosures measured at each time point

Prior to the nutrient additions, the NO_3_^−^ and PO_4_^3−^ concentrations in the two natural melt ponds were very similar, being 0.75–0.85 and 0.10–0.11 µmol L^−1^, respectively. The NO_3_^−^ addition increased the concentrations in the enclosures to 27 and 25 µmol L^−1^ in the single and dual nutrient enclosures, respectively (Fig. 3a). Similarly, the PO_4_^3−^ concentrations in the enclosures increased to 4.3 and 5.3 µmol L^−1^ (Fig. 3b). The concentrations of NO_3_^−^ and PO_4_^3−^ in the natural melt pond rose to 40.9 and 6.6 µmol L^−1^, respectively (Fig. 3c, d). In all the treatments, the nutrient concentrations decreased rapidly (Fig. 3), but while the low nutrient values persisted in the enclosures, the concentration in the natural pond declined below the detection limit after 7 days of incubation (Fig. 3). However, the concentration of bromide tracer in the enclosures remained stable during the 7–13 days of incubation (Fig. 4). Analyses of the Br^−^ concentration in the control enclosure showed a decrease by 50% on day 7, which was likely related to a short, albeit unobserved, submersion. Bromide was not added to the natural melt ponds. The initial salinity was 2.2 psu and showed an average increase of 0.10 ± 0.02 psu per day in the enclosures (*n* = 4), indicating slight evaporation, while the concurrent increase in both of the natural melt ponds was 0.20 psu per day (data not shown). The experiments to determine the sorption of PO_4_^3−^ onto aluminum pipes demonstrated a linear PO_4_^3−^ depletion over the days, which we attribute to a binding to the aluminum hydroxide coating (data not shown; Parfitt 1978). The rate of the PO_4_^3−^ sorption was 9.1 nmol cm^−2^ day^−1^, while the NO_3_^−^ concentrations remained unaffected. Scaled to the aluminium pipe surface area and the enclosure water volume, this sorption could account for 49% of total PO_4_^3−^ depletion in the enclosure with only PO_4_^3−^ addition and 65% in the enclosure with dual nutrient addition.

### Temporal variation in Chl *a*, particulate organic carbon (POC) concentration

**Fig. 5:**
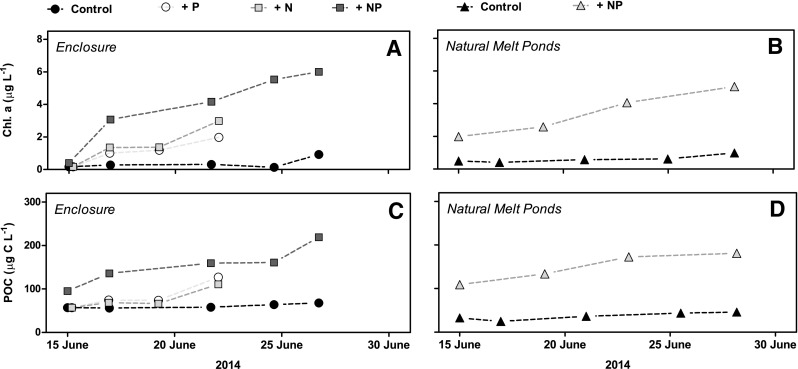
Increase in Chl *a* concentrations in **a** enclosures and **b** natural melt ponds during the study and associated increase in POC concentrations in **c** artificial and **d** natural melt ponds. *Connect lines* between the *symbols* does not represent the slopes used in the statistics, but are only present to increase readability

The Chl *a* concentration increased significantly (*p* ≤ 0.040) in all enclosures and in the two natural melt ponds, except in the enclosure with single addition of NO_3_^−^ where the linear trend was not significant due to a large scatter of the data points (*p* = 0.064) (Fig. 5a, b). During the 13-day incubation period, the rate of Chl *a* accumulation was 10-fold higher in the enclosure with dual nutrient addition compared with the control (slopes differed, *p* < 0.0001, Fig. 5A, B), while the increase in the enclosures with PO_4_^3−^ addition was intermediate with a ~fivefold increase during 7 days of incubation (*p* = 0.010). In the natural melt pond with nutrient addition, the increase in Chl *a* concentration was sevenfold higher than in the control pond (*p* < 0.0001, Fig. 5b). Similar to the Chl *a* concentrations, POC concentrations reflected nutrient additions in both the enclosures (Fig. 5c) and the natural melt ponds (Fig. 5d), with significant increases in all enclosures and natural melt ponds (*p* ≤ 0.049). In general, the POC concentrations correlated linearly with Chl *a* values, with a Chl *a*:POC ratio of 0.03 (µg Chl *a*/µg C, *p* < 0.0001).

### Nutrient-induced changes in primary and bacterial production

**Fig. 6:**
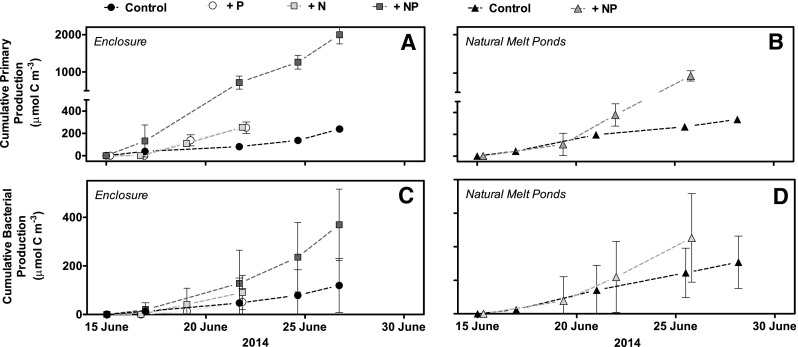
Cumulative primary production (*n* = 3) measured during the study in **a** enclosures and **b** natural melt ponds, with complementary cumulative bacterial production (*n* = 4, **c, d**). The primary and bacterial production rates were extrapolated to cumulative rates using the short-term incubations to integrate over the entire study and thereby obtain the accumulated photosynthetic biomass according to time points. *Error bars* indicate standard error (SE), which was estimated by time integrating the standard deviations from the separate short-term incubations and taking the sum of these. All measurements contain *error bars*, but some are smaller than the symbol size. *Connect lines* between the *symbols* does not represent the slopes used in the statistics, but are only present to increase readability

The ^14^C-based primary production rates from the short-term incubation were cumulated over the entire study to represent the production of photosynthetic biomass over time. Primary production rates increased significantly in all treatments and controls (Fig. 6a, b, p ≤ 0.012). The cumulative primary production was, however, ninefold higher in the enclosure with the dual nutrient addition compared with the control enclosure after 13 days of incubation (slopes differed, *p* < 0.0001 Fig. 6a). The treatments with the addition of either NO_3_^−^ or PO_4_^3−^ showed intermediate increases with threefold higher levels after 7 days of incubation compared with the control (*p* ≤ 0.0046, respectively, Fig. 8a). The increase in cumulative primary production was only twice as high in the natural melt pond with nutrient addition compared to the control pond (*p* < 0.0001, Fig. 6b). Collectively, the cumulative primary production (µmol C L^−1^) was positively correlated with the Chl *a* (µg L^−1^) concentration (slope 0.21 ± 0.03, *p* = 0.003).

**Fig. 7:**
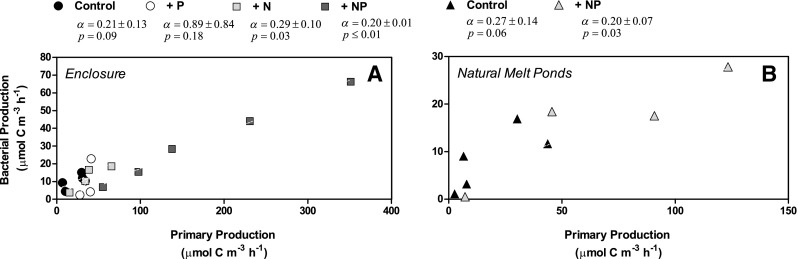
Bacterial production rates plotted against primary production rates from both **a** enclosures and **b** natural melt ponds, showing a positive correlation, with slope ± SE displayed for each

The cumulated bacterial production rates tended to increase in all treatments, but the increase was only statistically significant at dual addition of nutrients after 13 days (*p* = *0.014)*. The independent measurements of bacterial production using thymidine or leucine tracers resulted in relatively similar rates with an average difference of 17%, which provides confidence in the applied approaches as discussed in Chin-Leo and Kirchman (1988; below 25% difference). In both the enclosures and the natural ponds, the cumulative bacterial production closely reflected the pattern observed for the cumulative primary production (Fig. 6c, d), with a positive linear relationship between the primary and bacterial production when compiling all treatments and controls (*p* values = 0.0004, Fig. 7a, b).

### Melt pond algal and grazing community, ciliate biovolume and higher trophic levels

**Fig. 8:**
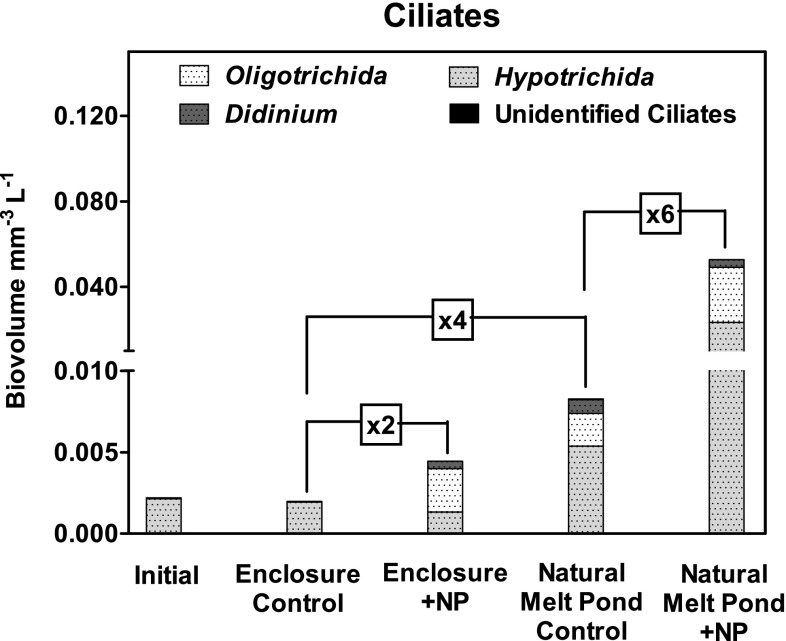
Changes in ciliate biovolume (mm L^−1^) in two of the enclosures and two natural melt ponds during the entire study, including the controls and the melt ponds with both NO_3_ and PO_4_^−^ additions. The “initial” samples represent the starting value for both the enclosures and the natural melt ponds, while the remaining are samples retrieved at the end of the study. The identified ciliate sub-species are marked with different colors: *Oligotrochida* sp. (*white*), *Hypotrichia* sp. (*light grey*), *Didinium* sp. (*dark grey*) and few unidentified (*black*). The difference in total biovolume (mm^−3^ L^−1^) between samples is indicated with *connecting lines*

The algal communities in both the melt ponds and enclosures were dominated by diatoms (*Nitzschia* sp., *Fragilariopsis* sp., and *Navicula* sp.) and an unidentified bi-flagellate (~5 µm). No changes in the community structure was observed in the enclosures, but in the natural melt ponds the dominant diatom genera had shifted to *M. arctica, Pseudonizschia* sp. and *Coscinodiscus* sp. suggesting recruitment of algae from the ice during the study period. The grazing communities were completely dominated by protozoans. Three ciliate types were identified: *Oligotrichida* sp., *Hypotrichida* sp. and *Didinium* sp., and only few ciliates could not be assigned to a specific clade (less than 1% of the total). Single specimens of the rotifer *Polyarthra* were identified in the control natural melt pond and in the final samples from the enclosure with nutrient addition (one organism in each), while the shell from a calanoid nauplius was identified in the natural melt pond with nutrient addition. No other potential grazers were observed. The total ciliate biovolume (mm^3^ L^−1^) in both the natural melt ponds and in the enclosure with dual nutrient addition increased (no replicate was sampled) during the study period, while the ciliate biovolume remained constant in the control enclosure (Fig. 8). Compared with the control, the increase in ciliate biovolume was two- and sixfold higher in the enclosure and natural melt pond with nutrient addition after 13 days of incubation, respectively (Fig. 8). Furthermore, the increase in ciliate biovolume was four times higher in the natural melt ponds than in the enclosures, suggesting recruitment of ciliates from the ice (Fig. 8).

### Sea ice productivity below snow cover

**Fig. 9:**
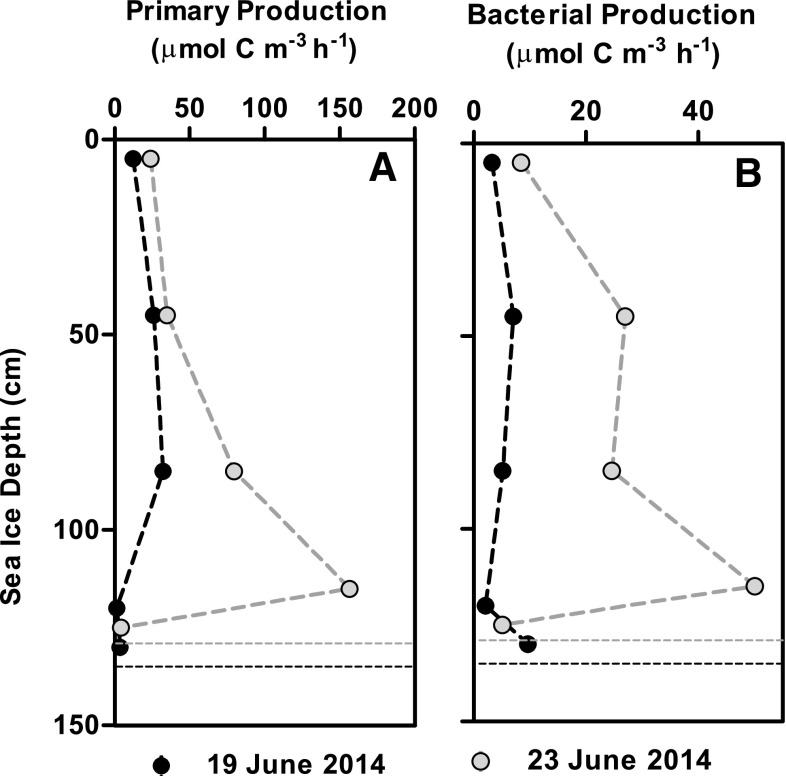
**a** Primary and **b** bacterial production rates in sea ice based on PE relations from five different sea ice depths measured on two occasions. Production rates and sea ice height from each sampling session are indicated with *circles* and *dotted lines*, respectively, *black* indicating measurements from 19 June and *grey* measurements from 23 June

Primary and bacterial production rates were determined in snow-covered sea ice on 19 and 23 June. The depth-specific production rates were highest on the latter date presumably due to a reduction in snow cover from 19 to 14 cm, which increased the light availability. From 19 to 23 June, depth-integrated primary production rates were 0.6 and 2.0 mmol C m^−2^ day^−1^, while the depth-integrated bacterial production rates amounted to 0.2 and 0.7 mmol C m^−2^ day^−1^, respectively. Overall, the highest rates of both primary and bacterial production were observed towards the bottom of the sea ice, the highest rates being measured in the 80–90 cm section on 19 June and in the bottom 10 cm on 23 June (Fig. 9a). The bacterial production (µmol C m^−3^ h^−1^) was linearly related to primary production (µmol C m^−3^ h^−1^) with an overall slope of 0.27 ± 0.04 (bacterial/primary production, *p* = 0.0024).

## Discussion

### Melt pond versus sea ice primary productivity

Only few studies have assessed primary production in melt ponds and to our knowledge none of these have included bacterial production and quantification of biomass at the higher trophic levels. Without nutrient addition, primary production rates in the natural melt ponds corresponded, on average, to 0.44 ± 0.38 mmol C m^−3^ day^−1^, resembling the rates of 0.02–0.68 mmol C m^−3^ day^−1^ reported in melt ponds on sea ice floes in the Canadian basin (Lee and Whitledge 2005). Thus, the coastal location of our study, with the higher potential for enhanced supply of air-borne material and nutrients from the relatively dry adjacent landmasses, had no clear effect on productivity as measured in the study. Additionally, the primary production rates of the melt ponds were comparable with those measured in the upper 10 cm of the sea ice at in situ light conditions, amounting to 0.30 (19 June) and 0.58 mmol C m^−3^ day^−1^ (23 June). Although the volumetric rates in the melt ponds were similar to those from the surface ice, the relatively small volume of melt ponds compared with the total sea ice volume implied that the melt pond production was insignificant relative to that of the sea ice. This was apparent when comparing depth-integrated primary production rates from the melt ponds (0.07 mmol C m^−2^ day^−1^) with those from the sea ice (0.64–1.98 mmol C m^−2^ day^−1^). Hence, our study confirms that melt pond productivity is low relative to that of sea ice (Mundy et al. 2011; Lee et al. 2012), except under conditions promoting excessive occurrence of algal biomass in the form of aggregates or mats (e.g. Lee et al. 2011; Fernández-Méndez et al. 2014). We can, however, not exclude potential loss of bacterial biomass due to osmotic stress during the thawing procedure and this could potentially have affected bacterial production (Miller et al. 2015).

### Nutrient limitation for melt pond primary production

Our study showed that Chl *a* concentrations and primary production rose with nutrient addition (Figs. 5a, b, 6a, b), the most noticeable difference occurring when adding both PO_4_^3−^ and NO_3_^−^. Based on observations of nitrogen depleted-conditions in investigated melt ponds, Mundy et al. (2011) suggested that NO_3_^−^ can be the limiting nutrient for melt pond primary production, which is also considered to be the case for the Arctic Ocean in general (Tremblay at al. 2015). However, our observations indicated that both NO_3_^−^ and PO_4_^3−^ availability stimulated productivity in melt ponds and that the combined addition provided the highest level of primary production. However, neither micronutrient and silicate were included in the investigations and could potentially have further stimulated the production. This finding reflects the recently acknowledged consensus that both nitrogen and phosphorous are needed for optimal production (Elser et al. 1990; Arrigo 2005). The phenomenon is referred to as co-limitation of nutrients, and data compilations in both Elser et al. (2007) and Harpole et al. (2011) have shown that this is a naturally occurring phenomenon in marine, limnic as well as terrestrial systems. This is particularly evident in limnic systems (Elser et al. 2007), supporting that co-limitation is likely to occur in melt ponds as these can occasionally be considered as limnic systems. This co-limitation is mainly explained by the fact that these systems mostly host a variety of different species whose individual members are limited by only one single nutrient (Arrigo 2005; Harpole et al. 2011). The physiological diversity of the algae community in the melt ponds may therefore, explain the observed co-limitation.

The concentration of added nutrients declined surprisingly fast (Fig. 3), faster than what could be accounted for through primary production. Assuming Redfield stoichiometry, only 2–26% of the nutrient decline could be ascribed to primary production (Table 1). Since this estimate is based on gross primary production it would also, for a first approximation, include the amounts of nutrients subsequently transferred to bacterial and ciliate biomass. It was neither raining nor snowing during the study period, which otherwise could have caused dilution of both the enclosures and natural melt ponds. Dilution from snow melt and drainage through sea ice brine channels may explain the loss of nutrients in natural melt ponds (Eicken et al. 2002), but this could not be the case for the enclosures. Here, exchange was prevented, and the Br^−^ data documented that there was limited exchange with the surrounding ice. The adsorption of PO_4_^3−^ onto the aluminum hydroxides coating the pipes, as determined in laboratory experiments, corresponded to 49 and 65% of the total PO_4_^− 3^ depletion in the enclosures with the single addition of PO_4_^3−^ and the enclosure with dual nutrient addition, respectively. Adsorption onto the pipes may, therefore, explain most of the fast decline in PO_4_^3−^. However, the large removal of NO_3_^−^ and part of the PO_4_^3−^ decline remain unexplained. Some diatoms have been shown to intracellularly accumulate NO_3_^−^ concentrations up to ~275 mmol L^−1^ (Kamp et al. 2011), and C:N ratios as low as 3.8 have been observed in nitrate replete cultures (Lomas and Gilbert 2000). This likely explains a fraction of the NO_3_^−^ depletion during our study, as the dominating algal species were diatoms. Thus, the nitrate uptake may have been approx. twice as high as estimated from the Redfield ratio in Table 1. Similarly, a luxury uptake of PO_4_^3−^ of up to 4 times the growth requirement has been observed in microalgae (Powell et al. 2011). Such an uptake could explain the missing PO_4_^3−^ in the enclosure with the dual nutrient addition, but not in the enclosure with the single addition of PO_4_^3−^ (Table 1). Another potential explanation for the NO_3_^−^ and PO_4_^3^ depletion could be formation of biofilm on the surface of the plastic foil of the enclosures, but no biofilm development was observed (visual inspection only) suggesting that no extensive biofilm development occurred in the enclosures. Furthermore, we cannot exclude the possibility of minor settlement of organic material, but it is not likely that accumulation of detritus can explain the missing nitrogen. Nevertheless, it is important to emphasize that despite the unexplainable high NO_3_^−^ depletion rate, our study showed a higher increase in primary production with the addition of nutrients compared with the controls, which was the main purpose of the investigation.

**Table 1:** The fraction of the added nutrients incorporated into algal biomass as estimated from cumulative primary production (µmol C m^− 3^) at the end of the study assuming a Redfield ratio for C/N/P of 106:16:1

	Incorporated fraction of added N (%)	Incorporated fraction of added P (%)
Enclosure (+N)	9	–
Enclosure (+P)	–	2
Enclosure (+NP)	26	9
Natural (+NP)	8	3

### Bacterial production and carbon demand

Bacterial production in the natural melt ponds (0.20 ± 0.14 mmol C m^−3^ day^−1^) was comparable with the volume-based rates recorded in the surface 10 cm sea ice (0.07 and 0.19 mmol C m^−3^ day^−1^, Fig. 9). Bacterial production was clearly correlated with the rates of primary production, which was presumably the main source of labile organic material sustaining the bacterial community in the melt pond water. This corresponds with the findings of previous studies linking bacterial production with primary production from both pelagic (e.g. Hoppe et al. 2002) and sea ice samples (Søgaard et al. 2010). The bacterial production rates in the controls equals to 44–47% of the primary production, while it amounted to 20–25% of the primary production with the addition of both NO_3_^−^ and PO_4_^3−^. This corresponds to values of most aquatic systems generally ranging about ~30% (Del Giorgio and Cole 1998; Cole et al. 1988). Converting the bacterial production into bacterial carbon demand (BCD) assuming a bacterial growth efficiency of 0.5 (e.g. Rivkin and Legendre 2001; Berggren et al. 2010; Nguyen and Maranger 2011), the BCD in the controls equals to 88–96% of the primary production. This suggests a tight coupling between primary production and bacterial growth. With the nutrient addition, the BCD only corresponded to 34–63% of the primary production in both the enclosures and the natural melt ponds, which might reflect a temporal decoupling from the increase in the primary relative to the bacterial production. Such decoupling has previously been observed in sea ice in the seasonal study by Nguyen and Maranger (2011).

### The effect of nutrient addition on grazers and higher trophic levels

In our study, ciliates appeared to be the only important grazers and they were shown to increase in number during the incubation period. While algae and bacteria are the main food source for *Oligotrichida* sp. and *Hypotrichida* sp., respectively, *Didinium* sp. predates on other ciliates (e.g. Verni and Gualtieri 1997; Hadas et al. 2014). *Didinium* sp. therefore, represents an additional trophic level in the investigated melt ponds along with the two identified rotifers (*Polyarthra* sp.). However, considering that only two rotifers were found in one of the natural melt ponds and one of the enclosures, rotifers do not appear to be important in the targeted melt ponds. The increase in ciliate biovolume was linked to the availability of organic carbon, ciliate biovolume becoming larger in the melt ponds with nutrient addition. This is consistent with findings by Gradinger et al. (1999) of enhanced ciliate concentrations with increasing availability of carbon recorded in a sea ice profile from the Greenland Sea. Based on a carbon content of 110 fg µm^−3^ (Königs and Cleven 2007), the estimated content of organic carbon in the ciliate biomass corresponded to only 0.1% of the POC in the enclosures, while the proportion was 4% in the natural melt ponds. The elevated proportion of ciliate biomass in the natural melt ponds compared with the enclosures, despite lower food availability, suggests recruitment of ciliates from the sea ice brine system. This is supported by the fact that ciliates are frequently observed in sea ice brine (Gradinger et al. 1999) as well as melt ponds (Bursa 1963) and that nutrient were presumably drained from the nutrient amended melt pond through such interconnecting brine channels. Overall, increased nutrient availability enhanced primary and bacterial production, which subsequently increased the numbers of ciliates in the sea ice melt water. Hence, nutrients stimulate productivity at several trophic levels.

Overall, sea ice melt ponds are short-lived ecosystems with a tight coupling between primary and bacterial production but with several trophic levels. Adding nutrients clearly increased production at several trophic levels, confirming that melt pond productivity is indeed limited by nutrient availability. Considering that the algal community in the melt ponds was predominantly diatoms (*Nitzschia sp., Fragilariopsis* sp. and *Navicula* sp.), silicate likely have a limiting effect (Brzezinski 1985; Fernández-Méndez et al. 2015), and addition of silicate may thus potentially have stimulated production even further. Hence, nutrient enrichment in the melt ponds may result in enhanced biological production and establishment of complete microbial ecosystems in melt ponds. Enrichment of nutrients in melt ponds can be induced by several mechanisms. Extensive snow loads on the sea ice can cause sea water flooding of the sea ice, whereby nutrient from surface waters can be transferred to the sea ice surface. Increased wind forcing can potentially promote both sea water spray from surface waters and terrestrial dust disposal from the relative dry landmasses. In addition, bird droppings and animal activity could cause a more random enrichment pattern on the sea ice surface. As shown by this study, nutrient enrichment through either of these sources are likely to enhance productivity on several trophic levels in the sea ice melt ponds.

UV radiation could be another important factor regulating biological production and trophic coupling in melt ponds. We did not investigate such potential effects during our study, but the UV levels were presumably the same in all targeted ponds and enclosures. UV radiation has previously been shown to inhibit primary producers (e.g. Marcoval et al. 2007; Wängberg et al. 2008), and a strong photoprotective response in the form of accumulation of carotenoid pigments has been reported in melt ponds by Mundy et al. (2011). A similar inhibitory effect of UV radiation has also been recorded for both bacterial production (e.g. Wängberg et al. 2008) and ciliate biomass (Marangoni et al. 2006; Summerer et al. 2009). If the bacteria and grazers are more strongly inhibited by UV radiation than are primary producers, UV radiation may thus have a stimulating effect on net productivity in the melt ponds (Agustí et al. 2014; Garcia-Corral et al. 2014). In our study, primary production determined in bottle incubations may have been stimulated relative to in situ rates due to the exclusion of UV radiation in the incubation bottles (e.g. Worrest et al. 1980; Agustí et al. 2014). Although further studies are needed to evaluate the combined impact of UV radiation and the stimulating effect of nutrient enrichment, the increase in Chl *a* in ponds and enclosures over time in our study clearly demonstrated that despite potential UV radiation biological production was indeed limited by nutrients. Since the melt pond coverage is predicted to increase in the future (Nicolaus et al. 2012; Rösel and Kaleschke 2013), these systems could become more important for the sympagic carbon cycling in the Arctic.
